# Notes and News

**Published:** 1897-07-24

**Authors:** 


### Notes and News.

(Collected and Communicated.)

At the Horton Infirmary two new children's wards have been opened, the gift of Mr. and Mrs. Mewburn.

The Prince of Wales has appointed Sir William MacCormac, Bart. (President of the Royal College of Surgeons, England), and Alfred Downing Fripp (M.S. London, and F.R.C.S. England) to be Surgeons in Ordinary to his Royal Highness.

At Mansfield the foundation-stone has been laid of a new wing to the Mansfield and Woodhouse District Hospital. The cost of the extension is estimated at £1,520, which has been contributed in commemoration of the long reign.

Mr. H. Sherwood Norris, of the Condal Water Company, informs us that he is offering to supply the London hospitals with the water at "practically cost price"; and, further, that his company will contribute annually to the Prince of Wales' Fund a "royalty" of threepence per dozen bottles sold. We shall record with interest any result of this offer of which we are informed.

At the last fortnightly meeting of the Governors of the Richmond Asylum, Dublin, Dr. Norman, medical superintendent, reported that since the previous meeting twelve females patient and three nurses had been attacked with beri-beri. These, with the six cases which had occurred earlier, made a total of 21 cases which had appeared in the female department since the beginning of June, exclusive of some four or five which remained over from the epidemic of last autumn and winter. One patient when attacked had only been in the asylum six weeks, and one nurse also six weeks. The disease appears not to have been quite absent since 1894. It tends to get worse in summer, attaining its height in autumn and passing away in winter and spring. There have been as many as 70 patients laid down at one time by it in the Richmond Asylum, the cases coming from all parts of the house, but those attacked this year appear to have been all females. Among the causes leading to the persistence of the disease in this asylum overcrowding would appear to take a high place. Dr. Norman reports that there are now on the books 919 male and 907 female patients, making a total of 1,826 patients, these numbers being respectively 414, 312, and 726 above the nominal limits of the original legitimate accommodation.

The plague is reported to have broken out at Constantinople; and small-pox amongst the volunteers at Kusuman in Bechuanaland.

Surrey has not been long in establishing its Convalescent Home for Women. A house has already been opened at Bognor pending the erection of a permanent institution at Ramsgate.

A cottage hospital has just been completed at Kirkcudbright. It is the gift of Mrs. Hope, of St. Mary's Isle, and contains two wards and offices. Mr. and Mrs. Hope will be responsible for its maintenance, but the patients will be required to make small contributions.

Dr. Patrick Manson, who has just been appointed Physician and Medical Adviser to the Colonial Office, in succession to Sir Charles Gage Brown, K.C.M.G., is Physician to the Seamen's Hospital, Albert Dock branch, the only hospital in London where Oriental diseases are treated in any number.

The fund for re-endowing Guy's Hospital creeps steadily up. The amount needed is so large that really handsome gifts appear small by contrast. A legacy of £10,000 not long since and a donation of £3,000 last week must bring the sum collected up to nearly £200,000. The interest of the last gift goes to sustain beds in the newly reopened Victoria Ward.

The City of London Hospital for Diseases of the Chest, Victoria Park, is financially in an unsatisfactory position, and it was therefore a matter of great importance that exceptional care should be taken in filling up the vacancy caused by the death of the late secretary, Captain Storrar Smith. The committee have elected Mr. Henry T. Dudley Ryder, assistant secretary to the City Carlton Club, to fill the vacancy in question. It appears from his testimonials and application that Mr. Ryder was for some time House surgeon and resident assistant accoucheur at King's College Hospital, and that he was compelled by protracted illness to abandon the medical profession. Mr. Dudley Ryder has had no special experience in the duties of the office to which he has now been appointed, though he states that he is deeply interested in hospital work, and particularly anxious to be more actively engaged in it. The present condition of the Victoria Park Hospital will afford Mr. Dudley Ryder an excellent opportunity for the exercise of every ability and influence which he may possess. We shall watch the progress of his career at Victoria Park with great interest, and we can only hope that, despite his inexperience, he may be enabled to set this interesting hospital upon its feet and induce many good men to join the committee, which has become depleted of late from internal causes, to which it is not necessary to refer on the present occasion. We would urge the medical staff to remember the deservedly high reputation which this hospital has held in the past, and to assert their influence and prerogative to secure that for the future due deference shall be paid to their advice and wishes, or failing this that they shall take a suitable opportunity of moving the governors to bring about necessary reforms and changes, without which not only the progress, but the very existence of the hospital may be imperilled.

